# A new and more informative subtyping scheme for breast cancer based on co-expression of metabolic genes

**DOI:** 10.1016/j.gendis.2025.101589

**Published:** 2025-03-06

**Authors:** Chongren Pei, Yuzhe Zhang, Jun Xiao, Qing He, Wenyong Zhang, Ying Xu

**Affiliations:** a Systems Biology Lab for Metabolic Reprogramming, Department of Human Cell Biology and Genetics, School of Medicine, Southern University of Science and Technology, Shenzhen, Guangdong 518055, China; b The First Laboratory of Cancer Institute, The First Hospital of China Medical University, Shenyang, Liaoning 110001, China; c Computation and Knowledge Engineering of Ministry of Education, College of Computer Science and Technology, Jilin University, Changchun, Jilin 130012, China; d The Third People's Hospital of Shenzhen, Shenzhen, Guangdong 518112, China; e School of Medicine, Southern University of Science and Technology, Shenzhen, Guangdong 518055, China

Breast cancer (BC), the most prevalent cancer type in women worldwide, exhibits significant heterogeneity across individual cases. The existing subtyping schemes for BC are all based on the expression of a few marker genes or proteins, which could not well capture samples sharing common or similar biology, which has substantially limited their applications.^1^ We present a new classification scheme for BC samples based on predominantly co-expression patterns of metabolic genes at the genome scale using a suite of machine-learning methods, resulting in four classes. Our consideration is: i) Extensive metabolic reprogramming is observed in cancer and considered as a hallmark of cancer^2^; and ii) all abnormal behaviors exhibited by cancer tissue cells are the direct results of these reprogrammed metabolisms, which are induced to adapt to specific stressors in the cancer-promoting microenvironments.^3^ In addition, the classification of BC tissues based on genome-scale metabolic genes expressions can ensure the stability of the classification results. Compared with the current PAM50-based BC subtypes, samples in each of our four classes have more outstanding shared metabolic characteristics, clinical features, genomic alterations, tumor microenvironments, immune-cell infiltrations, immunotherapy responses, and chemotherapy sensitivities, making our classification results more informative and potentially more useful.

We classified the BC samples collected from TCGA databases using the clustering function of the R package MOVICS, consisting of ten clustering methods: iClusterBayes, SNF, PINSPlus, NEMO, COCA, LRAcluster, ConsensusClustering, IntNMF, CIMLR, and MoCluster, based on co-expressions of metabolic genes (Supplementary Methods; Table S1) plus additional omics data such as genomic, proteomic, and methylomic data. This led to four distinct classes: CS1, CS2, CS3, and CS4 (Fig. 1A). The overall clustering procedure is an iterative process, which refines the selection of genes whose expression serves as the main clustering feature. In the final round of clustering, 100 genes were selected from each class, which were up-regulated only in the class of the BC samples. The nearest template prediction algorithm^4^ was employed for this round of classification. When 100 uniquely up-regulated marker genes were selected for each cluster, clustering results showed the strongest stability (*p* < 0.001; Fig. S1A, B). We demonstrated, via the silhouette score analysis, that the four classes each exhibited high similarities among their member samples and considerable differences between samples from different classes (Fig. S1C, D). We conducted survival analyses on each of the four classes, which revealed more significant differences in survival time across different classes compared with the current PAM50-based subtypes (Fig. 1B, C). We also analyzed the clinicopathological features of the new classification (Fig. S2A). In addition, we compared our classes with the PAM50-based subtypes, and found that 91.04% of CS1 members were in LumA; 74.19% of CS2 members were in Basal; while 53.46% and 42.14% members of CS3 were from LumA and LumB, respectively, and CS4's members were from all four subtypes. It is noteworthy that the four classes versus the four subtypes are quite consistent across different tumor stages, including in the validation dataset (Fig. S2B, C), strongly suggesting that the metabolic characteristics are an intrinsic characteristic of tumor development and do not change significantly with the stage of tumor development. Taken together, our new subtyping of BC samples is based on distinct metabolic characteristics of individual samples and is highly stable with the progression of the disease.

**Figure 1:**
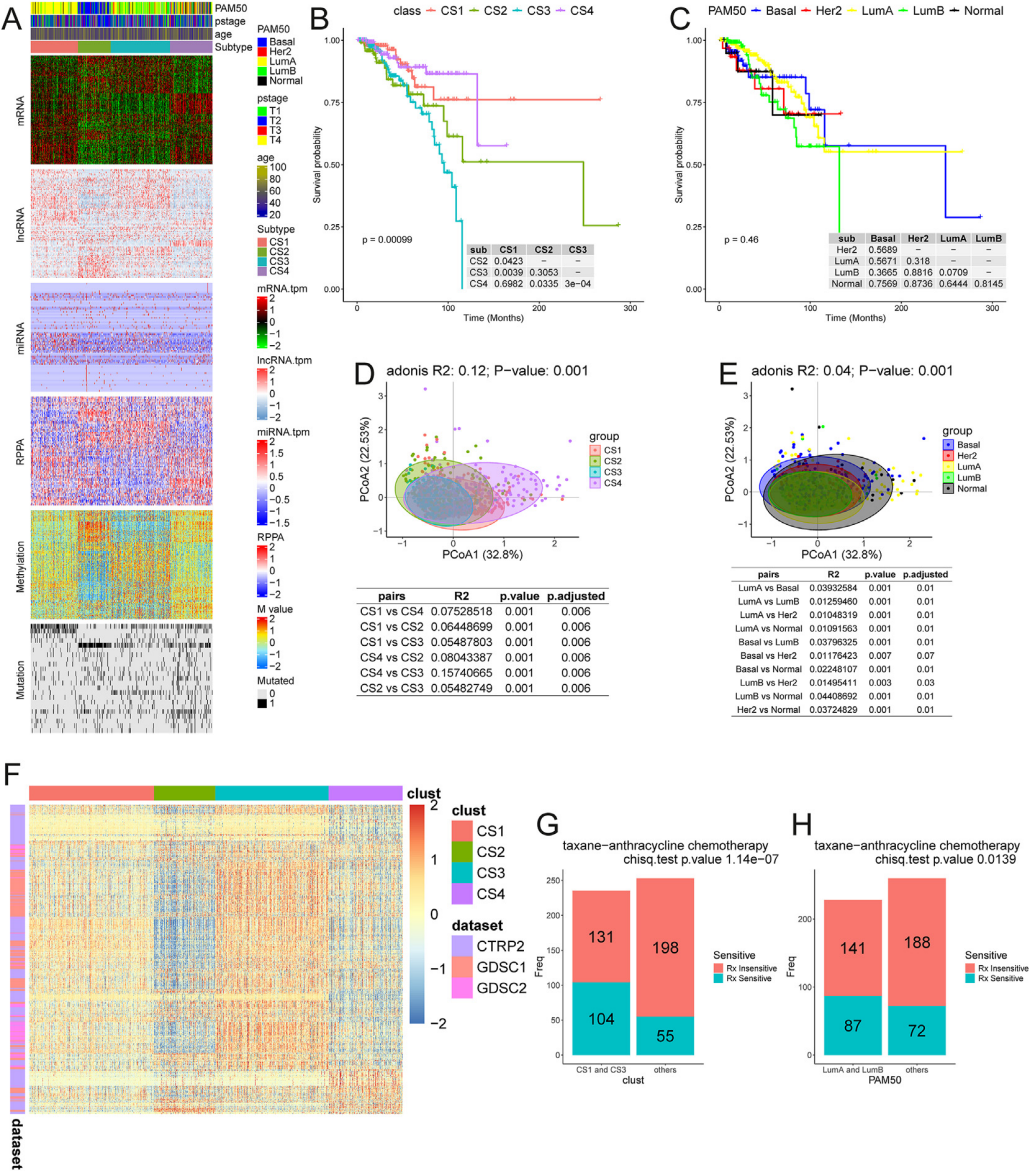
Four classes of breast cancer samples. **(A)** A heatmap for consensus clusters, consisting of levels of mRNAs of enzymes, lncRNA, miRNA, DNA CpG methylation sites, and mutated genes. **(B)** Kaplan–Meier survival curves for the four classes. **(C)** Kaplan–Meier survival curves for the PAM50-based subtype. **(D)** Similarities and differences in the immune microenvironments among the four classes. **(E)** Similarities and differences in the immune microenvironments among the four PAM50-based subtypes. **(F)** Heatmaps of IC50 values for drug sensitivity over samples in GDSC and CTRP databases. **(G)** Chi-square test for treatment effectiveness for CS1 and CS3 samples versus other classes in the GSE25066 dataset. **(H)** χ^2^ test for treatment effectiveness for luminal-A and luminal-B versus other classes in the GSE25066 dataset.

We analyzed the enzymatic genes that were differentially expressed in each class versus the other three using |log2(Fold Change)| ≥ 1 with *P*_adj_ < 0.05 as the criteria, followed with biological process enrichment analyses. Each class had its unique differentially expressed metabolic pathways, as summarized in Figure S3A. For more detailed information, we then conducted Gene Set Enrichment Analysis on each class of the BC samples together with the GSE202203 samples versus the other three classes, with the results summarized in Figure S3B–E. The main results were: i) CS1 had higher levels of metabolisms of medium and long-chain fatty acids, arachidonic acid, and butanoate; ii) CS2 displayed higher levels of metabolisms of sulfur compounds, one-carbon, glycoproteins, amino acids, glucose, and sphingolipids; iii) CS3 was characterized as having higher levels of metabolisms of insulin, hydroxyl compounds, catecholamines, long-chain fatty acids, oxidative phosphorylation, and *de novo* synthesis of nucleotides; and iv) CS4 was unique in its highly elevated immunological activities and metabolisms of prostaglandins, NAD^+^, tryptophan, and lipidolysis. Comparisons with PAM50-based subtypes are given in Figure S3F and G.

We showed that the top ten genes with the highest mutation rates in each class were greatly different across the four classes (Fig. S4A–D). First, the CS2 samples had the highest (average) tumor mutation burden, followed by CS4 and CS3, with CS1 having the lowest (Fig. S4E, F). Interestingly, none of the genes had high mutation rates, and each mutation occurred in only a few samples. To elucidate the functions of the mutant genes, we conducted pathway enrichment analysis over all the mutant genes in each class (Supplementary Methods). Table S2 lists the enriched pathways in each of the four classes. We showed that each of CS1, CS2, CS3, and CS4 had a distinct set of enriched pathways, namely, cell cycle regulation, DNA damage checkpoint, and DNA replication for CS1, development of the nervous system and regulation of neurogenesis for CS2, and epigenetic regulation, coagulation, and transcriptional regulation by RNA polymerase for CS3, while CS4 was enriched by cell cycle regulation, DNA replication, DNA repair, and ribosomal RNA transcription. These results indicate that it is not the individual mutated genes but instead the biological processes enriched by mutated genes that determine the phenotype. Compared with PAM50-based subtypes, our subtyping is more distinct in terms of genetic mutations, as detailed in Figure S4G and H. Furthermore, most miRNAs had fairly small differences in their expression across different classes (Fig. 1A).

Although the effectiveness of immunotherapy for BC is well recognized, the response by different patients varies significantly. To investigate the possible reasons, we compared the infiltration levels by different immune cells in each class (Fig. S5A–D) versus the levels of treatment effectiveness. We found that CS4 had both the highest immune score and dysfunction score^5^ among the four classes, the two of which tend to have the opposite effects on immune checkpoint inhibitor therapy, and CS2 and CS3 had relatively lower values in both. In addition, CS2 had the highest level of tumor mutation burden score (Fig. S4E), suggesting that it potentially has a more positive response to immunotherapy. Based on these, CS4 might not be suitable for immune checkpoint inhibitor therapy because of its high dysfunction score. In contrast, CS2 patients may benefit more from immunotherapy than the other classes, with its high tumor mutation burden scores and low Tumor Immune Dysfunction and Exclusion (TIDE) scores (Supplementary Methods), suggesting a lower likelihood of immune escape. As a comparison, the current PAM50-based scheme did not give this level of clean separation among patients of different subtypes (Fig. 1D, E).

We then examined the effectiveness of the chemotherapy drugs from the Genomics of Drug Sensitivity in Cancer (GDSC) and Cancer Therapeutics Response Portal (CTRP) databases by calculating the IC_50_ score of each drug over each class of patients (Fig. 1F). We conducted the following analyses to demonstrate that our classification scheme leads to classes having more consistent treatment results than the current subtypes of BC samples. The GSE25066 cohort has recorded patients' responses to taxan-anthracycline chemotherapy. Knowing that CS1 and CS3 are composed mostly of members of luminal-A and luminal-B and that they are mostly sensitive to the same chemotherapy drugs, we compared the predicted drug sensitivities by members of CS1 and CS3 to those of luminal-A and luminal-B. *χ*^2^ tests showed that the sensitivity and specificity by members of CS1 and CS3 to taxans-anthracycline were substantially more significant (*p* = 1.14e-07) than patients of subtypes luminal-A and luminal-B (*p* = 0.01385) (Fig. 1G, H).

In summary, we proposed a more robust and biologically meaningful BC classification to guide the treatment of BC, with members of each class showing substantially more consistent biological functions than the current PAM50-based subtypes.

## CRediT authorship contribution statement

**Chongren Pei:** Writing – review & editing, Writing – original draft, Visualization, Validation, Software, Project administration, Methodology, Investigation, Data curation, Conceptualization. **Yuzhe Zhang:** Methodology, Data curation. **Jun Xiao:** Methodology, Data curation. **Qing He:** Writing – review & editing. **Wenyong Zhang:** Writing – review & editing, Project administration, Methodology. **Ying Xu:** Writing – review & editing, Supervision, Resources, Project administration, Methodology, Funding acquisition, Formal analysis, Conceptualization.

## Data availability

The TCGA cohorts were downloaded from the TCGA website. The GSE202203 and GSE25066 cohorts are available from the GEO website. The classifier is available on https://github.com/852588956/Breast-cancer-typing-by-metabolic-genes.

## Funding

This work is supported by grants from the 10.13039/501100001809National Natural Science Foundation of China (No. T2350010, W2431059) and the Key University Laboratory of Metabolism and Health of Guangdong, 10.13039/501100012449Southern University of Science and Technology (China) (No. 2022KSYS007). This research was also supported by the Center for Scientific and Engineering Computing at Southern University of Science and Technology (SUSTech).

## Conflict of interests

The authors declared no competing interests.
