# Supplementary material for: A new and more informative subtyping scheme for breast cancer based on co-expression of metabolic genes
Source: Genes Dis. 2025 Mar 6;13(2):101589. doi: 10.1016/j.gendis.2025.101589 (PMC12664634; doi:10.1016/j.gendis.2025.101589)
Supplement: Multimedia component 1 [file mmc1.docx]

# **MATERIALS AND METHODS**

### **Study cohorts**

Our study is conducted on the transcriptomic data of 1,226 BC samples and 113 cancer-adjacent tissues from TCGA. Multi-omics data types, namely transcriptome, genome, methylome, proteome, and clinical data were obtained from the TCGA official website.^1^ Transcriptomic data are also collected from the GEO database,^2^ consisting of two BC datasets for validation: GSE202203^3^ with 3,207 samples, METABRIC^4^ with 2,509 samples, GSE20685 ^5^ with 327 samples, and GSE25066^6^ with 508 samples. The transcriptome and clinical data for these GEO samples were downloaded using the R package GEOquery.^7^

All human enzyme genes were obtained from the HumanCyc (see Supplementary Table 1).^8^ The transcriptome data underwent log2(tpm+1) transformation. The genomic data included single-nucleotide mutations and copy number variations.

### **Classification method**

Classification of the BC samples in the TCGA was done using the R package "MOVICS", based on transcriptomic (tpm), genomic, proteomic, and methylomic data, where transcriptomic data consist of mRNA (converted by log2), lncRNA, and miRNA; genomic mutation data cover all single-point mutations in each genome; and proteomic data are the Reverse Phase Protein Arrays.^9^

We have then used the "getElites" function in the MOVICS package to screen for characteristic genes by setting the parameter "mad" to 1,500 to screen for the top 1,500 genes with the largest variations; the parameter "cox" to screen for prognostic related genes (with P-value < 0.05) along each data dimension; and the parameter "freq" to 10% to screen the top 10% genes with the most frequent mutations.

Ten clustering algorithms are selected in the "getMOIC" function to conduct clustering analysis by selecting the following methods under “methodlist”: iClusterBayes, SNF, PINSPlus, NEMO, COCA, LRAcluster, ConsensusClustering, IntNMF, CIMLR, MoCluster. For other parameters, default values are used provided by the MOVICS package. Then the "getConsensusMOIC" function is used to integrate the results of different algorithms that maximizes the consensus across the ten classification results.

We then determine the number of clusters for the BC samples, using the "getClustNum" function in the MOVICS package. Prior knowledge is also applied, giving rise to four classes.

### **Differentially expressed genes**

Differential expression analyses are conducted for members of each class against controls using the R package DEseq2^10^ to identify class-specific differentially expressed genes. Genes with an absolute log_2_(FC) value > 1.0 along with P.adjust value < 0.05 are considered as differentially expressed.

### **Pathway enrichment analyses**

Differentially expressed genes are subject to pathway enrichment analyses against metabolic pathways retrieved from the KEGG^11^ database (<https://www.genome.jp/kegg/>) and the GSEA MSigDB website^12^ (<http://gsea-msigdb.org/gsea>). Differential genes were GSEA analyzed using the R clusterProfiler package based on the set of metabolic genes.^13^

### **Genomic mutation analysis**

The *random mutation rate* for a cancer class is defined as the total number of single-point mutations divided by the total length of the relevant genomes. A gene in a BC genome is considered selected for mutation if the number of mutations in this gene is at least three times the random mutation rate. The R package "maftools"^14^ is used to identify the top10 mutated genes in each genome.

### **Target gene of miRNA**

We found the target genes corresponding to mirnas from the mirDIP database^15^.

### **Prediction of immune cell infiltration in cancer tissues**

The R-packet CIBERSORT^16^ is used to estimate the relative abundance of each of the following immune cell types in a specified cancer tissue: B naive cells, B memory cells, plasma cells, CD8 T cells, CD4 T cells naive, CD4 memory resting T cells, CD4 memory activated T cells, T follicular helper cells, T regulatory cells (Tregs), gamma delta T cells, resting NK cells, activated NK cells, monocytes, macrophages M0, macrophages M1, macrophages M2, resting dendritic cells, activated dendritic cells, resting mast cells, activated mast cells, eosinophils, neutrophils based on the gene-expression data of BC tissues in the TCGA databases.

The R-packet ESTIMATE^17^ is used to predict the level of infiltration of stromal cells and immune cells in tumor tissues, and the two scores are added together to obtain the *estimate score*, which is used to infer the *tumor purity* of each tissue.

### **Prediction of immunotherapy response**

TIDE scores for specified cancer tissues were obtained through the TIDE website,^18^ where a higher TIDE score indicates that a cancer tumor has a strong immune-escape capacity, which is associated with a poor immune checkpoint suppression treatment response.

### **Assessment of similarity and difference between sample groups**

Multivariate nonparametric tests were analyzed using R package pairwiseAdonis^19^ based on Euclidean distance.

### **Drug sensitivity prediction**

We predicted drug sensitivity by samples in TCGA and GEO based on their transcriptome data using the R package oncopredict^20^ and drugs in GDSC^21^ and CTRP databases,^21,22,23^ which calculates the IC50 score for each sample. The lower an IC50 score, the more sensitive a cancer tissue is to the drug.

### **Statistical analysis and mapping**

All calculations were performed using R (version 4.3.2) for statistical analyses. Unpaired Student’s t‐test was applied for comparing two groups with normally distributed variables, while Wilcoxon test was utilized for non‐normally distributed variables. Overall survival (OS) analyses were conducted with survival package, with log‐rank test for determining survival difference. p < 0.05 indicated statistical difference.

**Supplementary references**

1. Tomczak K, Czerwińska P, Wiznerowicz M. Review<br>The Cancer Genome Atlas (TCGA): an immeasurable source of knowledge. *Contemp Oncol (Pozn)*. 2015;2015(1):68-77. doi:10.5114/wo.2014.47136

2. Clough E, Barrett T. The Gene Expression Omnibus Database. In: Mathé E, Davis S, eds. *Statistical Genomics: Methods and Protocols*. Springer; 2016:93-110. doi:10.1007/978-1-4939-3578-9_5

3. Dalal H, Dahlgren M, Gladchuk S, Brueffer C, Gruvberger-Saal SK, Saal LH. Clinical associations of ESR2 (estrogen receptor beta) expression across thousands of primary breast tumors. *Sci Rep*. 2022;12(1):4696. doi:10.1038/s41598-022-08210-3

4. Curtis C, Shah SP, Chin SF, et al. The genomic and transcriptomic architecture of 2,000 breast tumours reveals novel subgroups. *Nature*. 2012;486(7403):346-352. doi:10.1038/nature10983

5. Kao KJ, Chang KM, Hsu HC, Huang AT. Correlation of microarray-based breast cancer molecular subtypes and clinical outcomes: implications for treatment optimization. *BMC Cancer*. 2011;11:143. doi:10.1186/1471-2407-11-143

6. Hatzis C, Pusztai L, Valero V, et al. A Genomic Predictor of Response and Survival Following Taxane-Anthracycline Chemotherapy for Invasive Breast Cancer. *JAMA*. 2011;305(18):1873-1881. doi:10.1001/jama.2011.593

7. Davis S, Meltzer PS. GEOquery: a bridge between the Gene Expression Omnibus (GEO) and BioConductor. *Bioinformatics*. 2007;23(14):1846-1847. doi:10.1093/bioinformatics/btm254

8. Trupp M, Altman T, Fulcher CA, et al. Beyond the genome (BTG) is a (PGDB) pathway genome database: HumanCyc. *Genome Biology*. 2010;11(1):O12. doi:10.1186/gb-2010-11-s1-o12

9. Boellner S, Becker KF. Reverse Phase Protein Arrays—Quantitative Assessment of Multiple Biomarkers in Biopsies for Clinical Use. *Microarrays*. 2015;4(2):98-114. doi:10.3390/microarrays4020098

10. Love MI, Huber W, Anders S. Moderated estimation of fold change and dispersion for RNA-seq data with DESeq2. *Genome Biology*. 2014;15(12):550. doi:10.1186/s13059-014-0550-8

11. Kanehisa M, Goto S. KEGG: Kyoto Encyclopedia of Genes and Genomes. *Nucleic Acids Research*. 2000;28(1):27-30. doi:10.1093/nar/28.1.27

12. Liberzon A, Birger C, Thorvaldsdóttir H, Ghandi M, Mesirov JP, Tamayo P. The Molecular Signatures Database Hallmark Gene Set Collection. *Cell Systems*. 2015;1(6):417-425. doi:10.1016/j.cels.2015.12.004

13. Yu G, Wang LG, Han Y, He QY. clusterProfiler: an R Package for Comparing Biological Themes Among Gene Clusters. *OMICS: A Journal of Integrative Biology*. 2012;16(5):284-287. doi:10.1089/omi.2011.0118

14. Mayakonda A, Lin DC, Assenov Y, Plass C, Koeffler HP. Maftools: efficient and comprehensive analysis of somatic variants in cancer. *Genome Res*. 2018;28(11):1747-1756. doi:10.1101/gr.239244.118

15. Tokar T, Pastrello C, Rossos AEM, et al. mirDIP 4.1—integrative database of human microRNA target predictions. *Nucleic Acids Research*. 2018;46(D1):D360-D370. doi:10.1093/nar/gkx1144

16. Chen B, Khodadoust MS, Liu CL, Newman AM, Alizadeh AA. Profiling Tumor Infiltrating Immune Cells with CIBERSORT. In: von Stechow L, ed. *Cancer Systems Biology: Methods and Protocols*. Springer; 2018:243-259. doi:10.1007/978-1-4939-7493-1_12

17. Yoshihara K, Shahmoradgoli M, Martínez E, et al. Inferring tumour purity and stromal and immune cell admixture from expression data. *Nat Commun*. 2013;4(1):2612. doi:10.1038/ncomms3612

18. Jiang P, Gu S, Pan D, et al. Signatures of T cell dysfunction and exclusion predict cancer immunotherapy response. *Nat Med*. 2018;24(10):1550-1558. doi:10.1038/s41591-018-0136-1

19. Arbizu PM. *pairwiseAdonis: Pairwise Multilevel Comparison Using Adonis*.; 2017.

20. Maeser D, Gruener RF, Huang RS. oncoPredict: an R package for predicting in vivo or cancer patient drug response and biomarkers from cell line screening data. *Briefings in Bioinformatics*. 2021;22(6):bbab260. doi:10.1093/bib/bbab260

21. Yang W, Soares J, Greninger P, et al. Genomics of Drug Sensitivity in Cancer (GDSC): a resource for therapeutic biomarker discovery in cancer cells. *Nucleic Acids Research*. 2013;41(D1):D955-D961. doi:10.1093/nar/gks1111

22. Basu A, Bodycombe NE, Cheah JH, et al. An Interactive Resource to Identify Cancer Genetic and Lineage Dependencies Targeted by Small Molecules. *Cell*. 2013;154(5):1151-1161. doi:10.1016/j.cell.2013.08.003

23. Seashore-Ludlow B, Rees MG, Cheah JH, et al. Harnessing Connectivity in a Large-Scale Small-Molecule Sensitivity Dataset. *Cancer Discovery*. 2015;5(11):1210-1223. doi:10.1158/2159-8290.CD-15-0235

24. Rees MG, Seashore-Ludlow B, Cheah JH, et al. Correlating chemical sensitivity and basal gene expression reveals mechanism of action. *Nat Chem Biol*. 2016;12(2):109-116. doi:10.1038/nchembio.1986

**Supplementary figures**

**
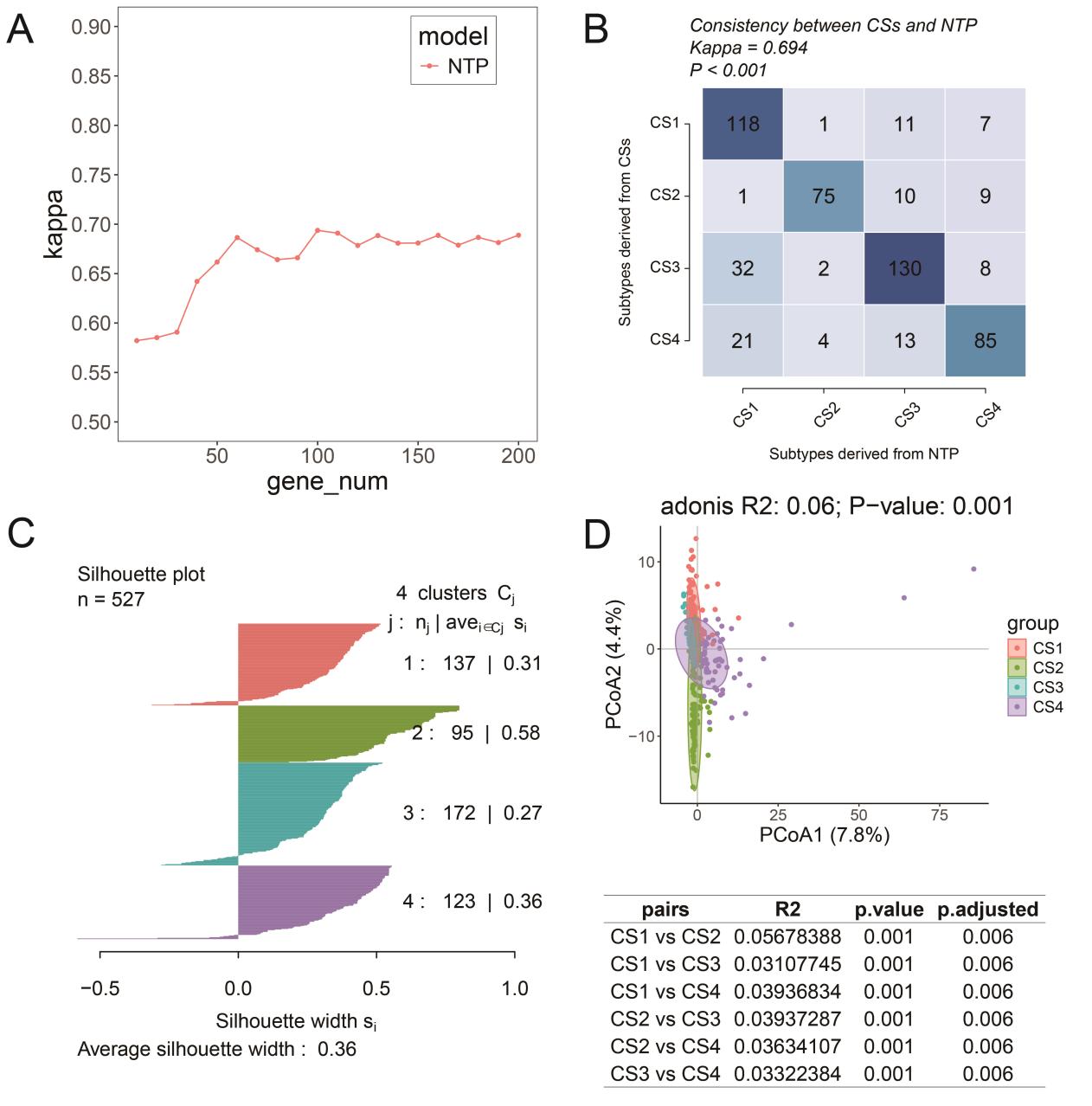
**

**Fig. S1: Stability of new metabolic classes.** (A) Kappa of 10-200 upregulated genes were selected for each class. (B) Consistency within each class with NTP in the training dataset. (C) Quantification of sample similarities using silhouette scores based on consensus clustering results. (D)The similarities and differences in terms of the metabolic characteristics among the four classes.


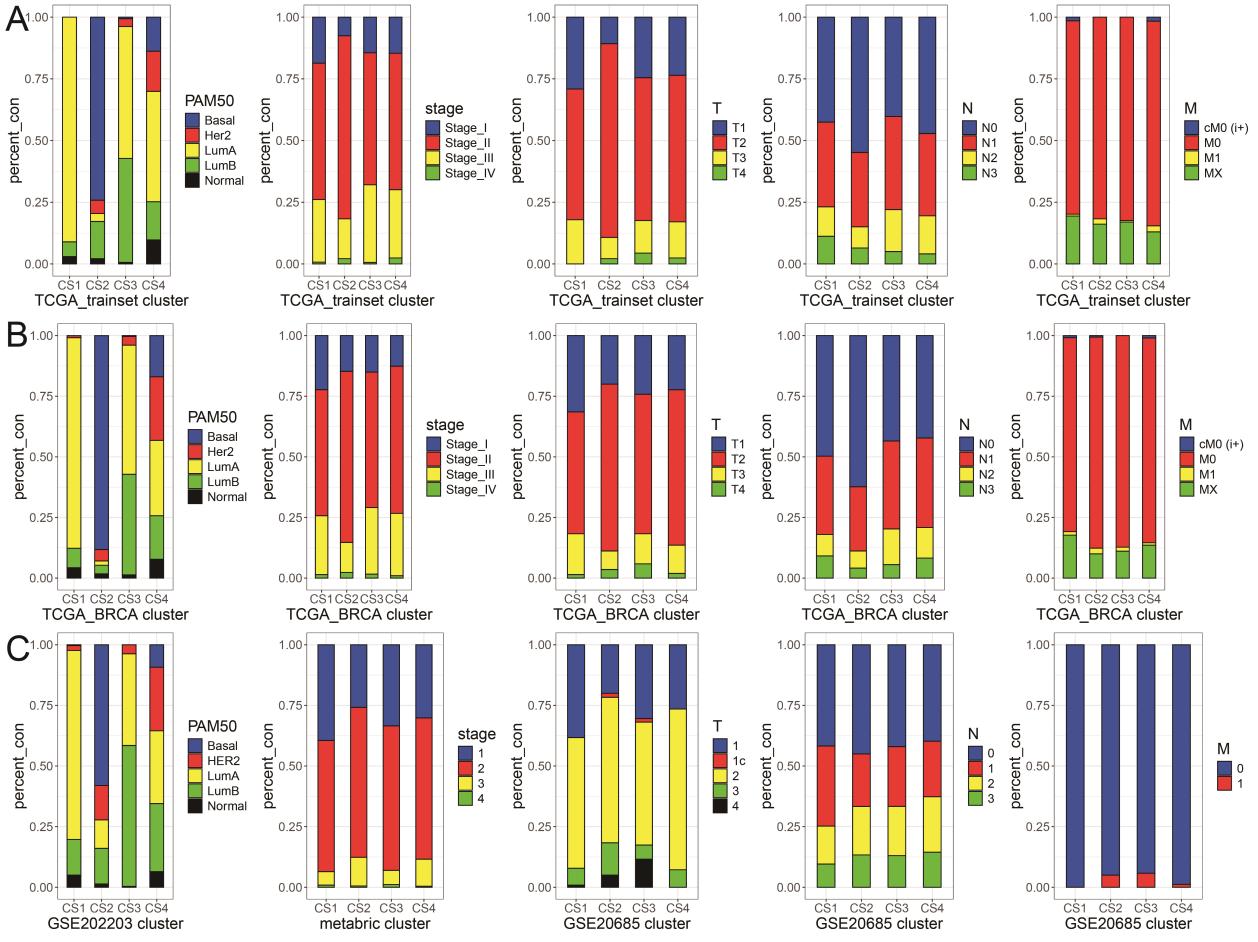


**Fig. S2: Distribution of clinical information in new metabolic subtypes.** (A) The proportion of the four classes in the PAM50 classification, tumor staging, and tumor-node-metastasis (TNM) classification of the training set. (B)The proportion of the four classes in the PAM50 classification, tumor staging, and tumor-node-metastasis (TNM) classification of the all BC samples of TCGA. (C) The proportion of the four classes in the PAM50 classification, tumor staging, and tumor-node-metastasis (TNM) classification of other BC data sets.


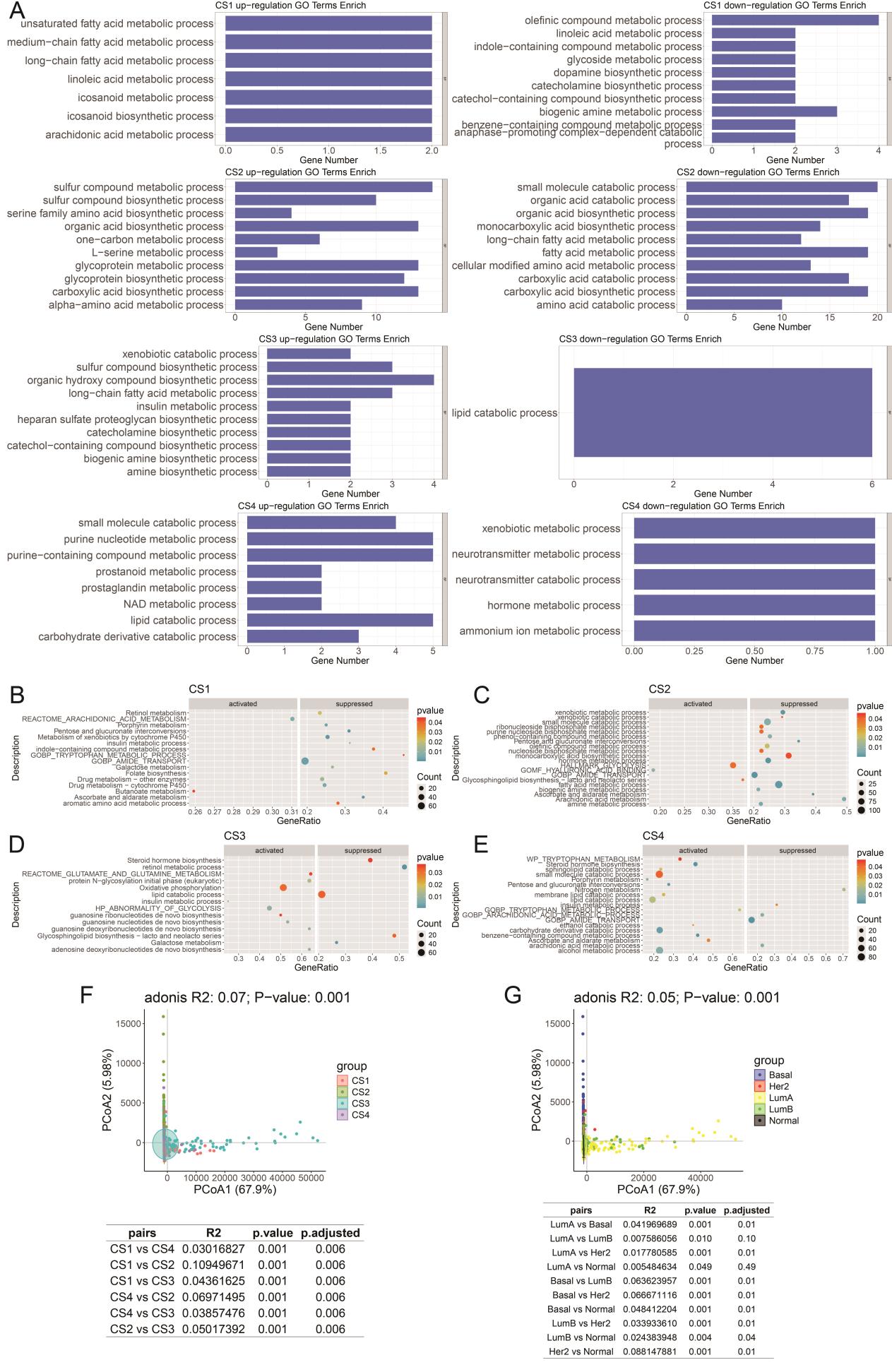


**Fig. S3: Enrichment pathways for new metabolic classes.** (A) Each class of uniquely upregulated gene and downregulated enriched metabolic pathways. (B-E) Outstanding metabolic pathways in each class. (F) Similarities and differences in the expression of metabolic genes among the four classes. (G) Similarities and differences in the expression of metabolic genes among the four PAM50-based subtypes.

**
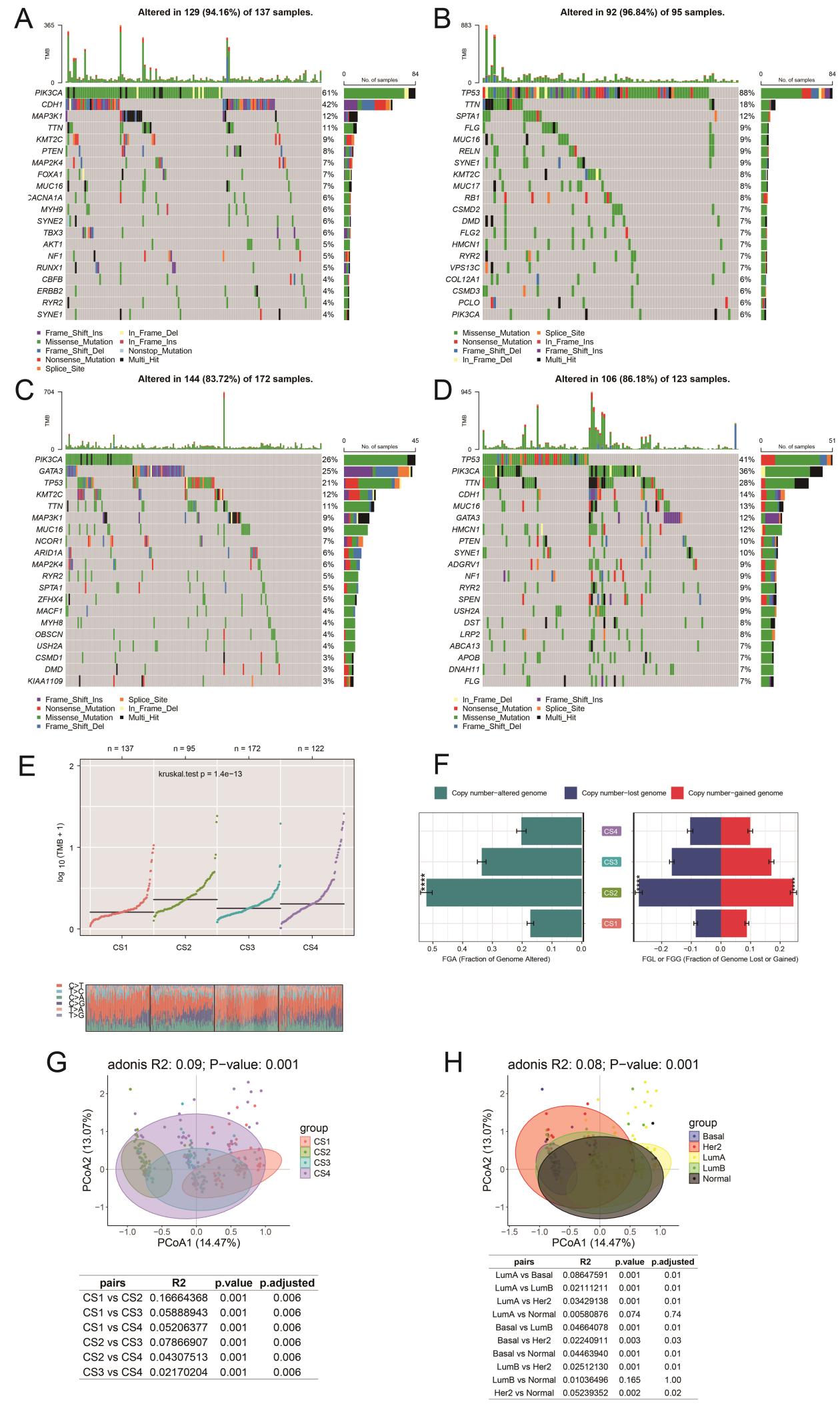
**

**Fig. S4**: Characteristic genomic mutations in the four classes of BC. (A-D) Ten most mutated genes in each of the four classes (OncoPrint). (E) Comparison of TMB and transitions and transversions in the four classes. (F) Copy number variation in four BC classes. (G) Similarities and differences in the gene mutation among the four classes. (H) Similarities and differences in the gene mutation among the four PAM50-based subtypes.


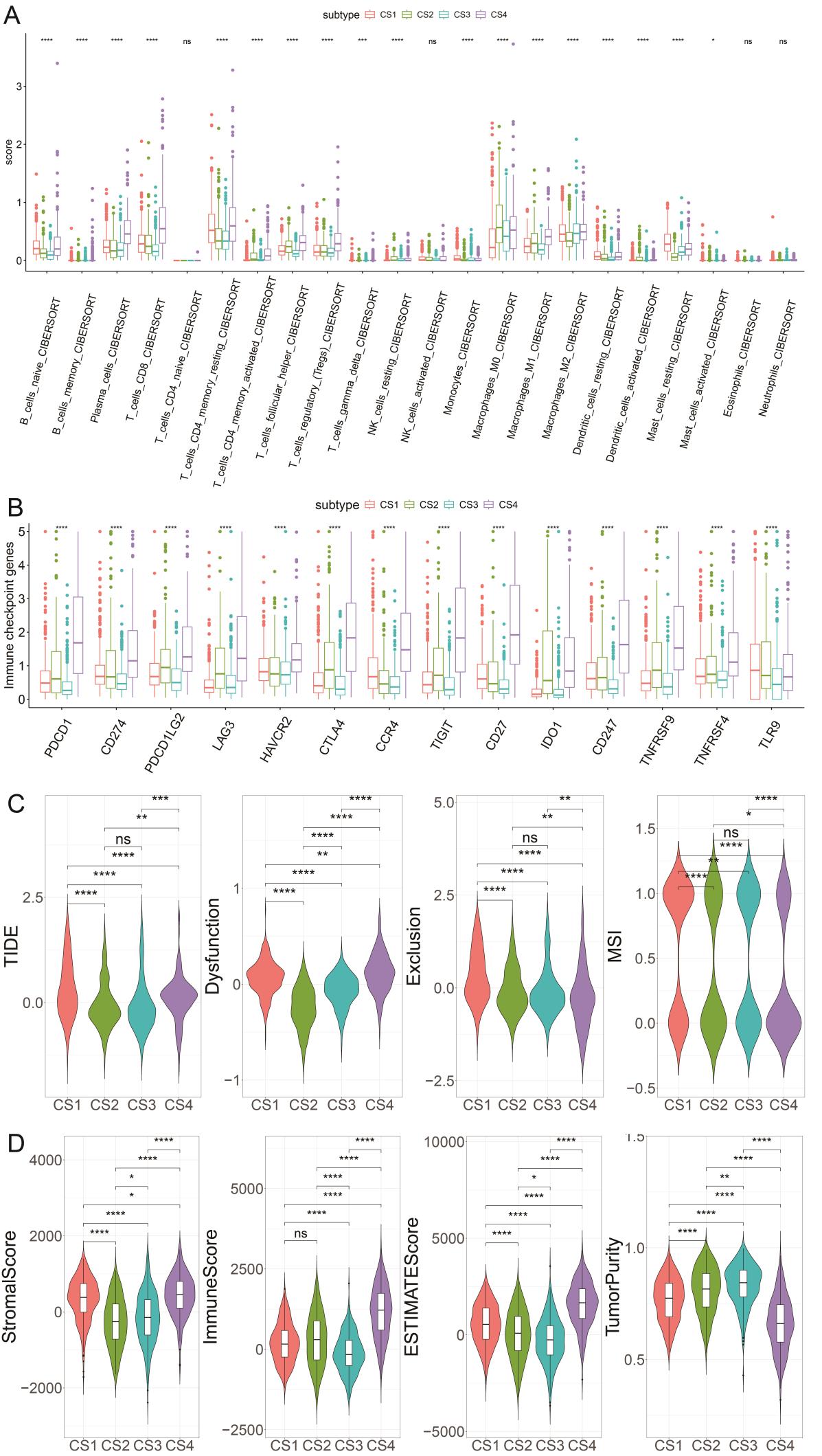


**Fig. S5:** Immune activities across the four classes. (A) Immune activities in each class. (B) Expression of immune checkpoints in each class. (C) Assessment of TIDE scores, dysfunction scores, exclusion scores, and microsatellite instability expression signature (MSI) scores for each class. (D) Evaluation of stromal scores, immune scores, combined stromal and immune scores, and tumor purity scores for each class.
